# Platform compliance governance in digital public health communication: effects of emergency health knowledge dissemination on protective behaviors

**DOI:** 10.3389/fpubh.2026.1904223

**Published:** 2026-07-31

**Authors:** Qing Lu, Haotian Chen, Yi Jiang, Shuhui Li, Shuaishuai Cao

**Affiliations:** 1 Communication School, Linyi University, Linyi, China; 2 College of Informatics, Harbin Institute of Technology, Shenzhen, China; 3 Law School, Peking University, Beijing, China; 4 Social Research Institute, University College London, London, United Kingdom; 5 Law School of Guizhou University, Guiyang, China

**Keywords:** digital public health, health information technology, health protective behaviors, knowledge absorption willingness, perceived compliance, platform governance, public health emergency knowledge

## Abstract

**Background:**

Digital health information platforms have become important channels for public health science communication and emergency prevention knowledge. However, uncertainty about content accuracy, information disclosure, privacy protection, and communication behavior may weaken public trust and reduce the conversion of health knowledge into protective behavior.

**Methods:**

Based on protection motivation theory and information adoption theory, this study constructed and tested a model linking public perceived compliance, knowledge absorption willingness, and health protective behaviors. Survey data were used to examine the associations between four dimensions of perceived compliance and health protective behaviors, as well as the mediating role of knowledge absorption willingness.

**Results:**

Perceived content compliance, information disclosure compliance, privacy protection compliance, and communication behavior compliance were positively associated with knowledge absorption willingness. Knowledge absorption willingness was positively associated with health protective behaviors and fully mediated the relationship between the four dimensions of perceived compliance and health protective behaviors.

**Conclusion:**

Platform compliance governance can support digital public health communication by strengthening users’ willingness to absorb health knowledge and convert it into protective behavior. The findings provide policy implications for health information quality control, privacy protection, and coordinated regulation in public health emergency knowledge dissemination.

## 1 Introduction

In the era of digital public health, short video platforms and other digital health information channels have become important infrastructure for public health science communication and emergency prevention knowledge. Their speed, reach, and visual presentation make them useful for translating professional health information into accessible knowledge. During the COVID-19 pandemic, such platforms helped communicate prevention measures and public health guidance to large audiences. At the same time, rapid content circulation also creates governance risks, including inaccurate science communication, incomplete source disclosure, insufficient privacy protection, and inappropriate promotional communication. These risks can weaken public trust and limit the behavioral effect of emergency health knowledge. It is therefore necessary to examine how perceived platform compliance shapes knowledge absorption and health protective behaviors in digital public health communication. This focus is consistent with the broader health policy concern of improving health information governance under digitalization and informatization.

This study focuses on four core issues: first, the current state of public perception regarding platform content compliance, information disclosure compliance, privacy protection compliance, and communication behavior compliance; second, the impact of different dimensions of perceived compliance on knowledge absorption willingness; third, the mediating role of knowledge absorption willingness between perceived compliance and health protective behaviors; fourth, proposed optimization strategies based on the research to enhance public health protective behaviors.

## 2 Literature review

Most existing research has focused on the respective connotations and individual impacts of public perceived compliance, knowledge absorption willingness, and health protective behaviors. It has mostly concentrated on general social media or online learning fields, but has insufficiently explored the mediating role of knowledge absorption willingness in the communication of public health science + emergency prevention and control knowledge on short video platforms and its influence mechanism on health protective behaviors.

### 2.1 Research on public perceived compliance

Public perceived compliance refers to the subjective judgment of the public on whether a platform’s communication behavior conforms to the standards of reliability, transparency, safety, and standardization based on their user experience and information exposure. Existing research mainly explores health-related social media use and platform communication from multiple dimensions, and these dimensions have confirmed that social media health information can influence users’ attitudes and behaviors.

The core of perceived content compliance lies in the public’s judgment of the scientific nature and authority. The positive impact of perceived legitimacy on rule compliance outweighs that of deterrent measures (1), providing a theoretical basis for compliance research. It should be noted that users’ cognition of the algorithm’s recommendation logic can enhance their perceived power and sense of internal control (2), increasing their compliance behaviors with the compliant content. Conversely, if the communication mechanism is opaque, it will weaken the perception of content compliance. In some regions, health decision-makers recognize the value of social media in health communication, but are also concerned about the accuracy of its content and the risk of misinformation (3). This practical demand further highlights the crucial role of content compliance in building a trustworthy communication environment and guiding health protective behaviors.

Perceived information disclosure compliance focuses on the impact of information transparency on public trust. The fairness of information exchange is a key driver of users’ willingness to disclose information (4). By clearly disclosing the qualifications of the content producers, data sources and applicable scope, platforms can reduce the public’s doubts about the authenticity of the information. Meanwhile, the assessment of the threat to the privacy of peers by users can affect their own privacy protection behaviors (5). Privacy self-efficacy and information security awareness play a moderating role, suggesting that platforms need to simultaneously explain the usage scope and protection measures for personal health information. Research based on COVID-19 vaccine information shows that risk disclosure has the greatest impact on public vaccination intention, and the persuasive effect of the loss framework is higher than that of the gain framework (6). While transparent disclosure of risks may cause concerns in the short term, it can enhance information trust in the long run.

Perceived privacy protection compliance refers to the public’s judgment on the security of platforms in protecting personal health information. Based on protection motivation theory, users’ assessment of the effectiveness of privacy protection mechanisms on social networks is positively correlated with their protection motivation. Privacy concern plays a mediating role between threat assessment and response assessment (7). That is, when platforms provide effective protection, the public is more willing to engage in health-related interactions and increase their willingness to pay attention to relevant knowledge, laying a foundation for the development of health protective behaviors. Privacy protection compliance is also an important component of perceived value. When the public perceives adequate protection measures, they consider that participating in health-related interactions brings more benefits than costs, and thus are more willing to engage in health-related interactions and in-depth learning (6).

Perceived communication behavior compliance focuses on the standardization and timeliness of the communication process. Therefore, inconsistent public health information can cause public confusion and distrust of health professionals, whereas timeliness and consistency can enhance compliance behaviors (8). Information that is presented in simple language and precisely targeted to high-risk groups is more easily understood and followed (9). It is suggested that the compliance of communication behaviors should meet the needs of the audience. Research on COVID-19 news and misinformation shows that exposure to misinformation is associated with weaker public health prevention, while credible public health communication can support preventive responses (10). This indicates that platforms’ review of the qualifications of the communication subjects and the content orientation directly affects the public’s trust in health protective knowledge and their absorption willingness.

### 2.2 Research on knowledge absorption willingness

Knowledge absorption willingness refers to the public’s subjective willingness to actively pay attention to, understand, integrate and apply knowledge. It serves as a crucial bridge connecting the public perceived compliance and their health protective behaviors. This study is mainly conducted from the following four aspects.

Active attention willingness refers to the public’s tendency to actively engage with health science knowledge on platforms. Health communication short videos can produce persuasive effects when content design and platform affordances fit users’ information needs (11). Therefore, platforms need to optimize content quality and recommendation algorithms to transform the public’s passive exposure to health knowledge into active attention. Evidence from short video platforms also shows that recommendation algorithms can shape users’ perceived stress and health management behavior (12), indicating that authoritative disease prevention content may attract active attention when users perceive it as valuable and reliable.

Deep understanding willingness is influenced by need–function fit. The higher the fit, the more easily the public can engage in immersive cognitive absorption (13). Meanwhile, for those with low prior knowledge, note-taking, explanations and other constructive activities can promote deep understanding. And for those with high prior knowledge, peer activities are more effective (14). This provides a reference for designing differentiated science popularization content on short video platforms, helping people with different levels of knowledge understand the key points of health protective behaviors.

The willingness to apply knowledge in real-life scenarios depends on the correlation between knowledge and life scenarios. Message-testing evidence from short-form health videos shows that concise, scenario-oriented video messages can encourage users to act on unfamiliar health information (15). Short video platforms, by simulating scenarios such as home epidemic prevention and allergy response, can help the public connect knowledge with real-life situations and clarify the operational methods of health protective behaviors. Furthermore, trusting beliefs influence social media users’ willingness to adopt and share health knowledge (16), thereby affecting the transformation of health knowledge into protective behaviors.

Sharing and communication willingness is driven by perceived value. Perceived value influences the willingness to share through the mediation of trust (17). When the public perceives that knowledge can help others or solve their own protection needs, the willingness to share increases. Meanwhile, attitude and perceived behavioral control have a significant impact on information sharing (18). This suggests that platforms need to enhance the credibility and practicality of the content to stimulate sharing and expand the communication scope of knowledge related to health protective behaviors.

### 2.3 Research on health protective behaviors

Health protective behaviors refer to practical actions related to health safety and emergency prevention and control after public exposure to health science popularization. These behaviors are significantly influenced by media communication, risk perception and social environment. They can be summarized into the following four dimensions.

The improvement of knowledge application behavior relies on active absorption and in-depth processing. When the public has a strong willingness to absorb knowledge, active deep learning can enhance the depth of mastery. Research on the role of constructive learning activities in promoting deep understanding (14) also supports this positive effect.

Risk judgment behavior is influenced by the compliance of information presentation. Research on vaccine information shows that risk disclosure and information framework affect vaccination intention (6), while misinformation in health crises can distort risk perception and compliance behavior (19), suggesting that short video science popularization needs to objectively disclose health risks to help the public accurately judge risk levels and thereby adopt scientific health protective behaviors, such as distinguishing between common cold and COVID-19. At the same time, compliant communication factors such as media exposure and framework are crucial for risk judgment (20).

The formation of active protective behaviors requires the establishment of the cognition that risks can be controlled. Research has found that individuals following the perception– controllability– high perceived risk pattern show high levels of cooperation and low levels of negative emotions (21). Short video platforms can help the public establish this cognition by providing timely and scientific compliant communication, guiding them to adopt clear and active protective behaviors, such as actively learning disinfection procedures and stocking emergency supplies.

The core of information communication trust behavior lies in the recognition of the platform and its content. Research on fake news detection has identified key factors of credibility such as source information and fairness (22). In health science popularization, public trust depends on the compliance of content, information disclosure, and communication behavior. When the platform provides authoritative content, transparent disclosure, and standardized communication, trust increases. Research on public health experts’ scientific communication to enhance information trust (9) also confirms that compliant communication is a core prerequisite for promoting the spread of health protective behaviors.

### 2.4 Summary

Existing research is characterized by multidisciplinary integration, solid theoretical foundation, empirical focus, and close alignment with reality. Especially in the context of COVID-19, related research has increased significantly. However, there are still some shortcomings: first, most studies focus on the direct effects of perception on behavior, while the mediating mechanism of knowledge absorption willingness is underexplored; second, the context is mainly focused on general social media or online learning, and there is a lack of research on health science popularization through short videos; third, the measurement dimensions of perceived compliance are not unified, and there is a lack of an integrated scale.

This study constructs a four-dimensional perceived compliance model covering content, information disclosure, privacy protection, and communication behavior, and verifies the mediating role of knowledge absorption willingness. Focusing on health science communication on Chinese short video platforms, such as Douyin and Kuaishou, the study provides targeted theoretical and practical guidance.

## 3 Research design

Although existing research has explored the connotations and individual impacts of public perceived compliance, knowledge absorption willingness, and health protective behaviors, in the specific scenario of public health science popularization + emergency prevention and control knowledge on short-video platforms, the interaction mechanism among these three factors, especially the mediating effect of knowledge absorption willingness, still lacks in-depth and systematic analysis. Moreover, the measurement dimensions of perceived compliance have not been unified, leading to insufficient contextual adaptability. To fill the aforementioned research gaps, this study adopts the following design:

### 3.1 Research hypotheses

The core independent variable of this study is the public perceived compliance of ‘public health science + emergency prevention and control knowledge’ communication on short video platforms. This refers to the public’s subjective judgment based on the user experience regarding whether the platform conforms to the standards of reliability, transparency, safety, and standardization in the four dimensions of content, disclosure, privacy and communication. The dependent variable is public safety cognition, which refers to the comprehensive cognitive state of knowledge application behavior, risk judgment behavior, active protective behavior and information communication trust behavior formed by the public after contacting platform knowledge. The mediating variable is knowledge absorption willingness, which refers to the subjective motivation intensity of public active attention, deep understanding, scenarios application and sharing and communication of knowledge. Terminology note: These expressions are related but not fully interchangeable. Public perceived compliance is used for the public-oriented construct, perceived platform compliance when the platform context is emphasized, and perceived compliance as a contextual shorthand when the meaning is clear.

#### 3.1.1 Perceived content compliance drives knowledge absorption

When the public determines that the short video content comes from authoritative sources such as the National Health Commission and the CDC, and is scientifically accurate without any exaggeration or false promotion, the valuable –reliable heuristic will be activated, significantly reducing the cost of information screening, and thereby enhancing the willingness to absorb knowledge (1). If the perceived content compliance is low, the useless –unreliable heuristic will be triggered, leading to knowledge avoidance. High willingness to absorb knowledge then promotes the deep processing and long-term memory of core knowledge by the public, ultimately improving the level of health protection (2). Therefore, this study proposes:

*H1*: Perceived content compliance has a significantly positive impact on knowledge absorption willingness.

*H1a*: Perceived content compliance has an indirect positive impact on health protective behaviors through knowledge absorption willingness.

#### 3.1.2 Perceived information disclosure compliance enhances knowledge credibility

When a platform clearly indicates the qualifications of the content producers, data sources, release time and applicable scope, it can significantly reduce the public’s uncertainty about the authenticity of the information, strengthening the information transparency –credibility judgment and thereby stimulating the public to invest cognitive resources to deeply understand the details of the knowledge and be willing to apply it to their own life scenarios. If the disclosure is incomplete, it is likely to trigger suspicion and cognitive withdrawal, weakening the willingness to absorb knowledge. As knowledge absorption willingness increases, the public becomes more capable of making accurate judgments regarding the risk levels of disease transmission and the effectiveness of prevention measures, and health protective behaviors are standardized (7). Therefore, this study proposes:

*H2*: Perceived information disclosure compliance has a significantly positive impact on knowledge absorption willingness.

*H2a*: Perceived information disclosure compliance has an indirect positive impact on health protective behaviors through knowledge absorption willingness.

#### 3.1.3 Perceived privacy protection compliance reduces psychological defense and promotes participation

When the public believes that the platform has implemented strict identity masking and encryption measures in health questionnaires, case sharing, and location services, they will experience a sense of privacy security–controllability. This significantly reduces their perceived risk of self-disclosure, making them more willing to actively engage in platform interactions, ask questions, and provide feedback, thereby enhancing their willingness to pay attention to knowledge and apply it in real-life scenarios. If perceived privacy protection is weak, it will trigger defensive avoidance, inhibiting knowledge absorption (6). High willingness to absorb knowledge prompts the public to convert the acquired knowledge into specific protective behaviors (such as home disinfection and emergency supplies preparation), and to form sustained trust in platform information (9). Therefore, this study proposes:

*H3*: Perceived privacy protection compliance has a significantly positive impact on knowledge absorption willingness.

*H3a*: Perceived privacy protection compliance has an indirect positive impact on health protective behaviors through knowledge absorption willingness.

#### 3.1.4 Perceived communication behavior compliance shapes platform credibility and sustains attention

In the event of a sudden outbreak of public health emergencies, if a platform can promptly forward official authoritative guidelines and clean up clickbait and malicious marketing content in a timely manner, it will strengthen the impression of standardized communication–responsible behavior and significantly increase the public’s attention and willingness to share the platform’s continuously updated content. If communication behaviors are improper, it will trigger the attribution of platform irresponsibility and lead to a decline in knowledge interest (10). With the increasing willingness to absorb knowledge, the public not only masters the correct operation process of health protection, but also is willing to spread authoritative science to family and friends for a second time, further consolidating and amplifying health protection through interaction (8). Therefore, this study proposes:

*H4*: Perceived communication behavior compliance has a significant positive impact on knowledge absorption willingness.

*H4a*: Perceived communication behavior compliance has an indirect positive impact on health protective behaviors through knowledge absorption willingness.

#### 3.1.5 Knowledge absorption willingness deepens health protective behaviors

High knowledge absorption willingness means that the public is willing to repeatedly watch complex tutorials, actively search for supplementary information, deeply integrate knowledge with their own life situations, and engage in cognitive reprocessing through sharing and discussion. This process not only enhances the accuracy of knowledge mastery, but also strengthens the clarity of risk judgment and behavior guidance, ultimately forming a high level of trust in platform information (10). Therefore, this study proposes:

*H5*: Knowledge absorption willingness has a significant positive impact on health protective behaviors.

#### 3.1.6 The mediating integration effect of knowledge absorption willingness

Public perceived compliance cannot be automatically transformed into health protective behaviors, and cognitive upgrading can only be achieved through a chain path of willing absorption– active processing– practical application. Only when the four dimensions of perceived compliance simultaneously or individually stimulate a strong willingness to absorb knowledge, will the public invest sufficient cognitive and emotional resources to achieve a transition from exposure to information to developing health protective behaviors (20). Therefore, this study proposes:

*H6*: Public perceived compliance has a significant positive impact on knowledge absorption willingness.

*H6a*: Public perceived compliance has a collective positive impact on health protective behaviors through knowledge absorption willingness.

### 3.2 Model construction

**Figure 1:**
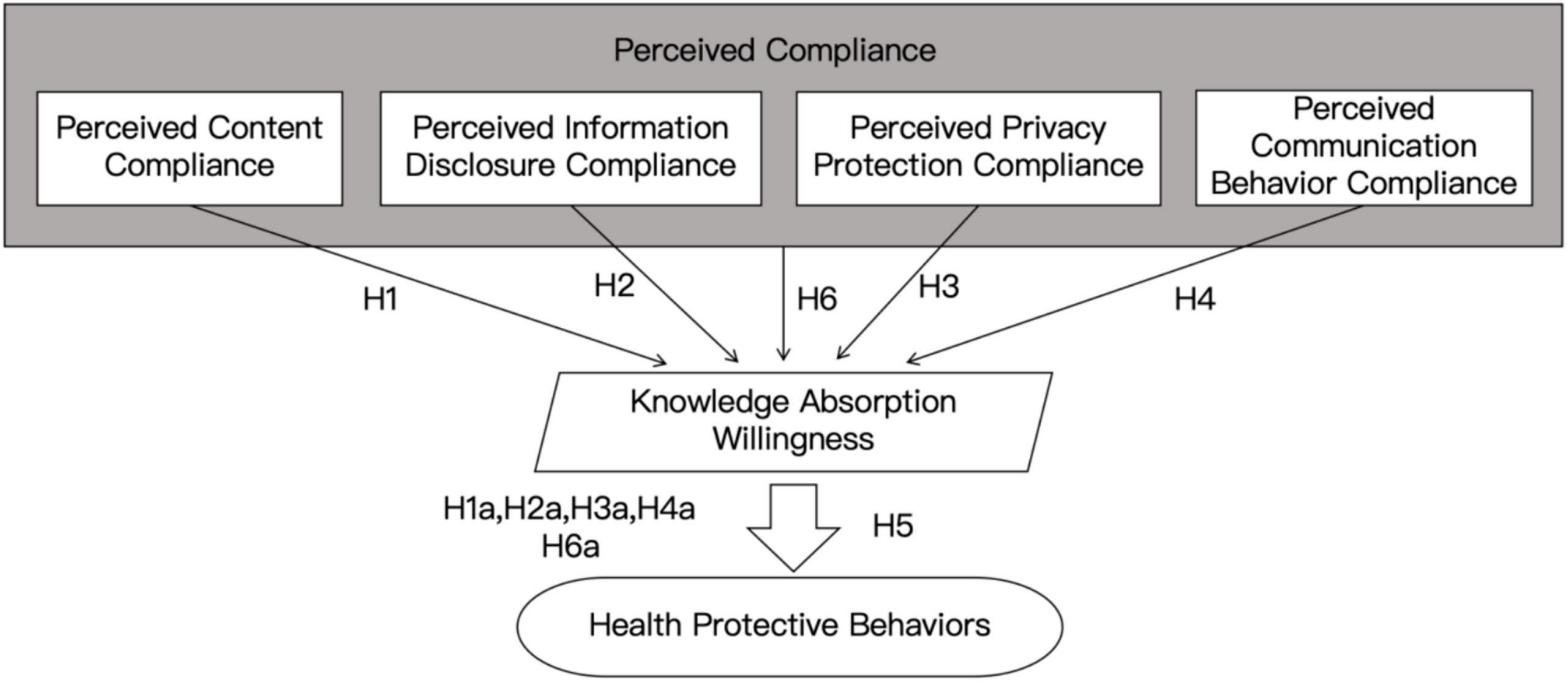
Schematic diagram of the influence mechanism of public perceived compliance on health protective behaviors in the context of public health science + emergency prevention and control knowledge communication on short video platforms.

Based on the theoretical logic and variable definition of public perceived compliance– knowledge absorption willingness– health protective behaviors, this study constructs an influence mechanism model for the communication effect of public health science popularization + emergency prevention and control knowledge on short video platforms. In the model, the independent variable public perceived compliance includes four dimensions: perceived content compliance, perceived information disclosure compliance, perceived privacy protection compliance, and perceived communication behavior compliance. The mediating variable knowledge absorption willingness has a positive impact on the dependent variable health protective behaviors, and the following model (Figure 1) is constructed:

### 3.3 Scale design and survey questionnaire sample selection

This study adopts a quantitative survey design. The core scales are grounded in protection motivation theory and information adoption theory and are adapted to the fragmented, scenario-based communication environment of short video public health science content. All items use a five-point Likert scale. The scale design emphasizes contextual fit, construct clarity, and internal consistency while retaining the substantive meaning of the original constructs.

#### 3.3.1 Scale design for public perceived compliance

This study is based on research hypotheses and model paths, dividing public perception into four dimensions and setting a total of 12 items. Each dimension is measured through three questions.

In the dimension of perceived content compliance, the scale design is based on information credibility theory, which points out that the authority of information sources and the scientific nature of content are the key supports of credibility. Regarding the scenario of health science popularization, although existing research has touched upon the perception of information legitimacy (1), there is insufficient attention paid to the professionalism and authority of health science popularization content. Another line of research focuses on the information compliance of algorithm recommendation scenarios (23), but the actual contextual suitability related to short video health science popularization and health protective behaviors is limited. This dimension combines the logic of threat assessment from protection motivation theory, and refers to relevant discussions on authoritative sources of health information (2). Based on the legitimacy of content sources, the fit between content and scientific knowledge, and the verification of content by medical experts, items are designed to systematically measure the subjective feelings of the public toward the compliance of health science popularization content, in order to fill the gap in contextual suitability and dimensional integrity of existing scales.

The construction of the perceived information disclosure compliance dimension is grounded in transparency theory, which emphasizes that information must be fully and clearly disclosed regarding sources, scope of application, and other key elements to enhance public trust. Although existing research has involved measuring information transparency (3–5), most of it focuses on social commerce scenarios and lack coverage of core elements such as data source labeling and indications of target populations for health protective knowledge in health science popularization. Or some studies focus on risk disclosure in vaccine information (5), but their contextual limitations are significant. Based on this, this dimension integrates the core viewpoints of information disclosure from the above research according to the logic of information quality assessment in information adoption theory. It adds items such as explanations of health data sources and indications of target populations for health protective knowledge. In doing so, it forms a measurement tool that better fits the context of health science popularization on short video platforms, providing transparent information support for health protective behaviors decision-making, and solving the problem of incomplete coverage of existing research dimensions.

The dimension of perceived privacy protection compliance is based on information security theory, which advocates for the protection of personal information from leakage or abuse through technological and institutional measures. Regarding privacy protection measurement in health scenarios, although existing research has designed privacy scales based on protection motivation theory (6), it has not involved the desensitization of personal sensitive information in patient cases. Other studies do not pay attention to the issue of third-party sharing of health data (7). To address this, this dimension is supported by the response assessment from protection motivation theory. It integrates relevant research conclusions on privacy protection and adds items such as sensitive information processing of patient cases and restrictions on third-party sharing of user health data, improving the measurement dimensions of privacy protection compliance, and filling the gap in existing scales for measuring health protection privacy.

The perceived communication behavior compliance dimension relies on communication content review theory, which holds that platforms need to ensure standardized and timely communication behavior through review mechanisms to avoid the spread of false or illegal content. Part of the findings focus on the timeliness of public health information (24), but do not address the efficiency of handling false content related to health protection. Some mention platform content review (9), but do not design measurement items for the induced marketing behavior in health science popularization. There is also a discussion on information communication focused on smoke events (8), which has strong contextual limitations. Based on this, this dimension is based on the logic of platform credibility assessment from information adoption theory and refers to relevant research on communication standardization. It adds items such as the efficiency of handling false health videos and the absence of induced marketing behaviors, thereby forming a more comprehensive measurement scale for perceived communication behavior compliance, addressing the issue of insufficient contextual relevance in existing research.

#### 3.3.2 Scale design for knowledge absorption willingness

The knowledge absorption willingness scale is designed based on the frameworks of knowledge absorption theory, deep learning theory, behavior change theory, and knowledge sharing theory. Among them, knowledge absorption theory emphasizes the attention, understanding, and application of knowledge, deep learning theory requires deep processing and internalization of knowledge, behavior change theory focuses on the influence of knowledge on health protective behavior tendencies, and knowledge sharing theory focuses on the transmission and diffusion of knowledge. Regarding the measurement of knowledge absorption willingness in the context of health science popularization, existing research has addressed active attention willingness (10, 12), but has not accounted for the characteristic of short video platforms where pushed content is viewed first. Some studies focus on a deep understanding of complex knowledge (14, 25), but have not considered the need for simplifying health protection terminology for easy understanding in short videos. Or only focus on the application of knowledge in general scenarios (15, 16), without covering health protection scenarios such as household emergency material preparation. At the level of sharing, it only focuses on knowledge sharing behavior (17, 18), but does not design items specifically for health protection science popularization content. Based on this, this scale takes the entire process of information reception–processing–application–communication from information adoption theory as the framework. It integrates the logic of knowledge absorption in each link of above research, optimizes the items according to the characteristics of short video health science popularization scenarios, and finally forms a measurement system with four dimensions of active attention–deep understanding–scenario application–sharing and communication. The scale consists of eight items, which help address the limitations of existing scales in terms of contextual suitability.

#### 3.3.3 Scale design for health protective behaviors

The health protective behaviors scale integrates knowledge application theory, risk judgment theory, active protective theory, and information communication trust theory: knowledge application theory focuses on individuals’ proficiency and structural understanding of knowledge, risk judgment theory focuses on individuals’ ability to identify and assess risks, active protective theory emphasizes the influence of attitudes and subjective standardization on behavior orientation, and information communication trust theory supports trust judgments on information. For research on knowledge application, existing studies focus on academic knowledge (14) and do not cover how to use health protective equipment. For research on risk judgment, although existing studies focus on public health events (20, 21), they do not cover the identification of health risks in real-life scenarios. For research on active protective behavior, studies of contradictory vaccine information on social media suggest that conflicting health content can interfere with protective judgment, but they do not fully cover the broader behavioral set of emergency health protection (26). For research on information communication trust, existing studies do not consider alignment with recommendations from the CDC (22). Research on social media health use, health-emergency misinformation, short-video information adoption, and fact-checking further informs the measurement boundary for information trust and sharing behavior (27–30). Therefore, this scale is based on the application–judgment–proactiveness–trust chain. Referring to the above research, 12 additional items such as the use of health protective equipment, risk identification in real-life scenarios, and alignment with recommendations from the CDC are added to improve the measurement dimensions of public health protective behaviors and solve the limitations of existing scales in measuring health emergency scenarios.

#### 3.3.4 Scale design for demographics and background information

In addition, the demographic and usage background items in the questionnaire strictly follow conventional demographic and behavioral measurement methods. These items cover five dimensions: age, gender, place of residence, educational level, and frequency of exposure to health science popularization knowledge. This ensures comprehensive collection of sample background information and provides a foundation for subsequent heterogeneity analysis.

Based on the scales above, this study’s questionnaire consists of 37 items in total.

### 3.4 Data collection and processing

This study used the online survey platform WJX for data collection. Because the study focused on public perceptions of real communication practices on short video platforms and their relationship with health protective behaviors, no experimental or control group was established. Respondents were asked to answer according to their actual experiences of encountering public health science and emergency prevention knowledge during daily short video platform use. This design improves ecological validity, but the results should be interpreted as survey-based associations rather than causal effects.

The questionnaire was distributed through social media, short video platform comment areas, online communities, and interpersonal sharing channels during 2025. Recruitment was adjusted for different age groups: for young respondents aged 18 to 35, distribution emphasized short video platforms and social media; for respondents aged 36 and above, recruitment was supplemented through family sharing and community groups. A total of 500 questionnaires were collected, and 476 valid questionnaires were retained after cleaning. This sampling strategy increased coverage across age groups, but it remains a non-probability online survey and cannot fully represent the general population.

## 4 Empirical analysis

### 4.1 Sample descriptive statistics

This study obtained a total of 476 valid questionnaires, accounting for 95.2% (based on the original collection of 500 questionnaires). As shown in Table 1, the frequency of the public’s exposure to public health science popularization or emergency prevention and control knowledge through short video platforms generally exhibits a low-frequency characteristic. Among them, more than half of the respondents (52.5%, *n* = 250) stated that they only encounter such content once or twice a month, making them the largest group. Another 17.6% (*n* = 84) of users are in a state of almost never encountering such content. The combined proportion of the two is as high as 70.1%, indicating that most users have relatively infrequent exposure to health and emergency-related information. In contrast, the proportion of users who truly achieve high-frequency exposure is extremely low: only 23.3% (*n* = 111) of users reach once or twice a day, while those who are exposed daily (including once or twice per day and three or more times per day) account for only 6.5% (*n* = 31) in total. From this, it can be seen that although short video platforms have become an important channel for information communication, the public’s active or passive exposure to public health and emergency prevention and control content is still insufficient. The overall frequency distribution shows an unbalanced feature of low-frequency dominance and high-frequency scarcity. This phenomenon may reflect the low weight of health science popularization content in algorithm recommendations, user interest orientation biased toward entertainment, or the need to improve content attractiveness and practicality. Therefore, how to enhance user stickiness through optimizing perceived compliance and content design is the key path to improving the effectiveness of public health information reach.

**Table 1:** Frequency distribution of respondents’ exposure to public health science popularization and emergency prevention and control knowledge via short video platforms.

Variable	Option	Frequency	Percentage	Mean	Standard deviation
Exposure interval	Almost never	84	17.6%	2.24	0.90
	Once or twice a month	250	52.5%		
	Once or twice a week	111	23.3%		
	Once or twice a day	4	0.8%		
	Three or more times a day	27	5.7%		

### 4.2 Reliability and validity testing

#### 4.2.1 Reliability testing

This study conducted reliability tests for the core latent variables. As shown in Table 2, the Cronbach’s alpha coefficients for most dimensions exceed 0.70, meeting the commonly accepted threshold in social science research (31). The Cronbach’s alpha for perceived privacy protection compliance is 0.614, which is lower than the conventional 0.70 threshold but may be acceptable for exploratory research (31). This result should be interpreted cautiously because privacy protection in digital health communication includes heterogeneous issues such as health information encryption, patient case de-identification, and third-party data sharing.

**Table 2:** Reliability coefficients of the short video scale.

Dimension	Number of items	Cronbach’s α
Perceived content compliance	3	0.747
Perceived information disclosure compliance	3	0.763
Perceived privacy protection compliance	3	0.614
Perceived communication behavior compliance	2	0.769
Knowledge absorption willingness	8	0.831
Health protective behaviors	12	0.885

#### 4.2.2 Validity testing

This validity analysis was conducted through exploratory factor analysis using SPSS software:

The results showed that the KMO value is 0.920, which was well above the recommended threshold of 0.80. This indicated strong partial correlations among the variables and a high degree of sampling adequacy, making the data highly suitable for factor analysis. At the same time, the Bartlett’s test of sphericity reached a significant level (χ^2^ = 2091.230, df = 55, *p* < 0.001), indicating a significant correlation between the items and demonstrating a high degree of structural validity in the data (Table 3).

**Table 3:** Validity testing of the scale.

Test	Statistic	Value
KMO measure of sampling adequacy		0.920
Bartlett’s test of sphericity	Approx. Chi-Square	2091.230
	df	55
	Sig.	0.000

### 4.3 Hypothesis testing

#### 4.3.1 Correlation analysis between variables

In order to enhance the reliability of the data, this study conducted correlation tests on the dependent variable, independent variable, and control variable. The correlation between variables is shown in Table 4. The study found that the perceived compliance across multiple dimensions (content, disclosure, privacy, communication) of short video platforms is significantly positively correlated with the knowledge absorption willingness and health protective behaviors of people over 65, with the role of information disclosure compliance being particularly prominent. Among them, the correlation coefficient between health protective behaviors and knowledge absorption willingness is the highest (r = 0.754, *p* < 0.001), indicating that the stronger the understanding and attention of the old populations to public safety information, the stronger their willingness to actively absorb health or emergency-related knowledge, and there is a highly synergistic effect between the two.

**Table 4:** Correlation between variables.

Variable	Perceived content compliance	Perceived information disclosure compliance	Perceived privacy protection compliance	Perceived communication behavior compliance	Knowledge absorption willingness	Health protective behaviors
Perceived content compliance	1					
Perceived information disclosure compliance	0.646***	1				
Perceived privacy protection compliance	0.536***	0.65***	1			
Perceived communication behavior compliance	0.64***	0.639***	0.502***	1		
Knowledge absorption willingness	0.554***	0.657***	0.562***	0.55***	1	
Health protective behaviors	0.544***	0.621***	0.62***	0.502***	0.754***	1

*p < 0.05, **p < 0.01, ***p < 0.001.

#### 4.3.2 Regression analysis of perceived content compliance on health protective behaviors

This section conducted a linear regression analysis on perceived content compliance and health protective behaviors, using SPSS to test the hypotheses. The regression results showed a linear relationship between the two, and the overall model was significant (*F* = 199.061, *p* < 0.001). Perceived content compliance has a significant positive impact on health protective behaviors. For every one-unit increase in platform content compliance, the level of health protective behaviors increases by an average of 0.458 units, with a 95% confidence interval of [0.395, 0.522]. Research has confirmed that strengthening the mechanism of source labeling and medical verification for short video content can effectively enhance users’ level of health protection, providing empirical support for platforms to optimize their health communication strategies (Table 5).

**Table 5:** Regression analysis results (independent variable: content = perceived content compliance).

Predictor	Coef.	St. Err.	t-value	*p*-value	95% Conf	Interval	Sig
Content	0.458	0.032	14.109	0	0.395	0.522	***
Constant	1.997	0.111	18.016	0	1.779	2.215	***

Mean dependent var	3.528
R-squared	0.296
F-test	199.061
Akaike crit. (AIC)	687.498
SD dependent var	0.591
Number of obs	476
Prob > F	0.000***
Bayesian crit. (BIC)	699.995

*p < 0.05, **p < 0.01, ***p < 0.001.

#### 4.3.3 Regression analysis of perceived information disclosure compliance on health protective behaviors

This section conducted a linear regression analysis on perceived information disclosure compliance and health protective behaviors, using SPSS to test the hypotheses. The regression results showed a significant linear relationship between the two, and the overall model was highly significant (*F* = 297.478, *p* < 0.001). Perceived information disclosure compliance has a significant positive impact on health protective behaviors (*β* = 0.406, *p* < 0.001), with a 95% confidence interval of [0.359, 0.452]. This means that for every one-unit increase in information disclosure compliance in health science popularization content (such as clearly indicating information sources, disclosing data sources, and stating the qualifications of the content provider), the level of health protective behaviors increases by an average of 0.406 units. The research results have validated hypothesis H2, indicating that enhancing the transparency and standardization of information disclosure, strengthening the mechanism of short video content source labeling and medical verification, can effectively improve users’ level of health protection, providing empirical support for platforms to optimize health communication strategies (Table 6).

**Table 6:** Regression analysis results (independent variable: disclosure = perceived information disclosure compliance).

Predictor	Coef.	St. Err.	t-value	*p*-value	95% Conf	Interval	Sig
Disclosure	0.406	0.024	17.248	0	0.359	0.452	***
Constant	2.242	0.078	28.923	0	2.090	2.394	***

Mean dependent var	3.528
R-squared	0.386
F-test	297.478
Akaike crit. (AIC)	622.538
SD dependent var	0.591
Number of obs	476
Prob > F	0.000***
Bayesian crit. (BIC)	635.034

*p < 0.05, **p < 0.01, ***p < 0.001.

#### 4.3.4 Regression analysis of perceived privacy protection compliance on health protective behaviors

This study conducted a linear regression analysis on perceived privacy protection compliance and health protective behaviors, using SPSS to test the hypotheses. The regression results showed a significant linear relationship between the two, and the overall model was highly significant (*F* = 296.748, *p* < 0.001). Perceived privacy protection compliance has a significant positive impact on health protective behaviors (*β* = 0.443, *p* < 0.001), with a 95% confidence interval of [0.392, 0.493]. When users perceive that the platform places more emphasis on personal information protection, data usage standardization, and privacy policy transparency in the process of health science popularization, their level of health protective behaviors increases by an average of 0.443 units. The research results support hypothesis H3, indicating that strengthening privacy protection compliance mechanisms not only concerns user rights, but also effectively enhances public understanding and awareness of public health risks. This provides key empirical support for short video platforms to embed a privacy-trusted design in health communication and to establish a positive cycle mechanism of privacy protection–health protective behaviors, emphasizing the dual value of balancing information transparency and user privacy protection in health communication (Table 7).

**Table 7:** Regression analysis results (independent variable: privacy = perceived privacy protection compliance).

Predictor	Coef.	St. Err.	t-value	*p*-value	95% Conf	Interval	Sig
Privacy	0.443	0.026	17.226	0	0.392	0.493	***
Constant	2.112	0.085	24.868	0	1.945	2.278	***

Mean dependent var	3.528
R-squared	0.385
F-test	296.748
Akaike crit. (AIC)	622.989
SD dependent var	0.591
Number of obs	476
Prob > F	0.000***
Bayesian crit. (BIC)	635.485

*p < 0.05, **p < 0.01, ***p < 0.001.

#### 4.3.5 Regression analysis of perceived communication behavior compliance on health protective behaviors

This study conducted a linear regression analysis on perceived communication behavior compliance and health protective behaviors, using SPSS to test the hypotheses. The regression results showed a significant linear relationship between the two, and the overall model was highly significant (*F* = 159.434, *p* < 0.001). Perceived communication behavior compliance has a significant positive impact on health protective behaviors (*β* = 0.358, p < 0.001), with a 95% confidence interval of [0.302, 0.414]. That is, when users perceive that the platform adheres to standardization in the process of health information communication (such as avoiding exaggeration, not creating panic, following scientific communication ethics, etc.), their level of health protective behaviors increases by an average of 0.358 units. The research results support hypothesis H4, indicating that standardized communication behavior mechanisms can effectively enhance public trust and security perception of health information. Standardized communication behavior is not only an extension of content compliance, but also an important guarantee for improving public risk cognition and scientific literacy. This provides empirical support for short video platforms to optimize health information communication behavior guidelines and verifies the theoretical hypothesis of behavioral compliance →cognitive reinforcement (Table 8).

**Table 8:** Regression analysis results (independent variable: behavior = perceived communication behavior compliance).

Predictor	Coef.	St. Err.	t-value	*p*-value	95% Conf	Interval	Sig
Behavior	0.358	0.028	12.627	0	0.302	0.414	***
Constant	2.253	0.104	21.733	0	2.049	2.457	***

Mean dependent var	3.528
R-squared	0.252
F-test	159.434
Akaike crit. (AIC)	716.382
SD dependent var	0.591
Number of obs	476
Prob > F	0.000***
Bayesian crit. (BIC)	728.879

*p < 0.05, **p < 0.01, ***p < 0.001.

#### 4.3.6 Regression analysis of public perceived compliance on health protective behaviors

This study conducted a linear regression analysis on public perceived compliance and health protective behaviors, using SPSS to test the hypotheses. The regression results showed a significant linear relationship between the two, and the overall model was highly significant (*F* = 431.512, *p* < 0.001). Public perceived compliance has a significant positive impact on health protective behaviors (*β* = 0.596, *p* < 0.001), with a 95% confidence interval of [0.539, 0.652]. That is, for every one-unit increase in users’ comprehensive perceived compliance with short video platforms across the dimensions of content, information disclosure, privacy protection, and communication behavior, their level of health protection increases by an average of 0.596 units. The research results have verified the core hypothesis, indicating that building a comprehensive and systematic platform compliance system is a key path to enhance public health risk cognition, providing strong empirical support for short video platforms to optimize the governance of health science popularization content and credible communication mechanisms (Table 9).

**Table 9:** Regression analysis results (independent variable: x = public perceived compliance; dependent variable: health protective behaviors).

Predictor	Coef.	St. Err.	t-value	*p*-value	95% Conf	Interval	Sig
Constant	1.566	0.096	16.227	0	1.376	1.755	***
Public Perceived Compliance	0.596	0.029	20.773	0	0.539	0.652	***

Mean dependent var	3.528
R-squared	0.477
F-test	431.512
Akaike crit. (AIC)	546.286
SD dependent var.	0.591
Number of obs	476
Prob > F	0.000***
Bayesian crit. (BIC)	558.782

*p < 0.05, **p < 0.01, ***p < 0.001.

#### 4.3.7 Hypothesis testing results

Based on the above analysis, the hypothesis testing results are shown in Table 10.

**Table 10:** Hypothesis testing results of public perceived compliance on health protective behaviors.

Research hypothesis		Supported or not
H	Public perceived compliance	Yes
H1	Content compliance	Yes
H2	Information disclosure compliance	Yes
H3	Privacy protection compliance	Yes
H4	Communication behavior compliance	Yes

The results in Table 10 indicated that all research hypotheses were supported. The comprehensive public perceived compliance had a significant positive predictive effect on health protective behaviors (*β* = 0.596, *p* < 0.001); all its sub dimensions also showed significant positive effects– content compliance (*β* = 0.458), information disclosure compliance (*β* = 0.406), privacy protection compliance (*β* = 0.443), and communication behavior compliance (*β* = 0.358), all at the *p* < 0.001 significance level. This indicates that the stronger users’ perceived compliance with platforms in terms of scientific nature of content, information transparency, data privacy protection, and communication ethics, the higher their level of understanding, judgment, and awareness of public health risks (i.e., health protective behaviors).

### 4.4 Mediation effect testing

To preliminarily examine the relationship between each compliance dimension and health protective behaviors, this study first conducted a linear regression analysis with the mean of the scale as the observation indicator. To further reveal the underlying mechanism and control measurement errors, a latent variable mediation model (SEM) was constructed for the core variables public perceived compliance, knowledge absorption willingness, and health protective behaviors. Due to different measurement methods, the effect sizes cannot be directly compared. In future research, latent scores may be uniformly used for full model estimation.

#### 4.4.1 Analysis of the impact of public perceived compliance on health protective behaviors through knowledge absorption willingness

The mediation analysis results showed that public perceived compliance had a significant positive impact on health protective behaviors, and this impact was mainly achieved through knowledge absorption willingness. The direct effect (c′ = 0.214, 95% CI [0.003, 0.438]) reached statistical significance (*p* < 0.05), but the indirect effect was more prominent: path a (public perceived compliance →knowledge absorption willingness, *β* = 0.893 ***) and path b (knowledge absorption willingness →health protective behaviors, β = 0.703 ***) were both highly significant. The indirect effect value was 0.628 (95% CI [0.456, 0.868]), and the confidence interval did not include zero (*p* < 0.01), indicating that knowledge absorption willingness plays a partial mediating role between the two variables. The total effect reached 0.841, indicating that perceived compliance strengthens health protective behaviors through both the direct path and the mediating path of knowledge absorption willingness. The public’s overall perception of compliance with health science popularization content on short video platforms not only directly enhances their health protection but also generates a stronger indirect effect by stimulating their willingness to absorb knowledge. The research results highlight the dual value of platform content compliance construction in public health communication– it not only enhances public trust, but also promotes knowledge internalization, thereby effectively strengthening risk response awareness (Tables 11, 12).

**Table 11:** Mediation analysis results - public perceived compliance.

Path	Estimate	95% CI [LL, UL]
Direct effect (c′)	0.214***	[0.003, 0.438]
Path a: Public perceived compliance → Knowledge absorption willingness	0.893***	[0.723, 1.114]
Path b: Knowledge absorption willingness → Health protective behaviors	0.703***	[0.52, 0.938]
Indirect effect (a × b): Public perceived compliance → Knowledge absorption willingness → Health protective behaviors	0.628**	[0.456, 0.868]
Total effect	0.841	—

c’ = direct effect; a x b = indirect effect; CI = confidence interval; LL = lower limit; UL = upper limit; *p < 0.05, **p < 0.01, ***p < 0.001.

The indirect effect is based on 5,000 bootstrap samples; **p < 0.01 (95% CI excludes zero).

**Table 12:** Mediation analysis results (lavaan, Bootstrap = 5,000).

Path	Estimate	95% CI [LL, UL]
Direct effect (c’)	0.282***	[0.205, 0.353]
Path a: X → M	0.712***	[0.644, 0.775]
Path b: M → Y	0.438***	[0.368, 0.515]
Indirect effect (a × b)	0.312**	[0.255, 0.375]
Total effect	0.594	—

c’ = direct effect; a x b = indirect effect; CI = confidence interval; LL = lower limit; UL = upper limit; * *p* < 0.05, ** *p* < 0.01, *** *p* < 0.001.

**p < 0.01 (95% CI excludes zero). Standard errors and CIs are based on 5,000 bootstrap samples.

The research results provide quantitative basis for the health science popularization strategy of short video platforms, emphasizing the dual importance of improving content compliance and enhancing users’ willingness to absorb knowledge. It has practical guidance value for optimizing the effectiveness of health communication.

#### 4.4.2 Analysis of the impact of perceived content compliance on health protective behaviors through knowledge absorption willingness

The mediation analysis results showed that the impact of perceived content compliance on health protective behaviors was primarily achieved through knowledge absorption willingness. The direct effect (c′ = 0.145, 95% CI [−0.022, 0.314]) did not reach statistical significance (confidence interval including zero), while the indirect effect was significant: path a (perceived content compliance →knowledge absorption willingness, *β* = 0.744 ***) and path b (knowledge absorption willingness →health protective behaviors, *β* = 0.759 ***) were both highly significant. The indirect effect value was 0.565 (95% CI [0.425, 0.754]), and the confidence interval did not include zero (*p* < 0.01), indicating that knowledge absorption willingness plays a fully mediating role between the two. The total effect was 0.71, indicating that although content compliance does not directly improve health protection, it can effectively stimulate the public’s willingness to absorb knowledge, thereby significantly enhancing their safety perception of public health risks. This finding emphasizes that ensuring scientific, accurate, and standardized content in short video health science popularization is a key mechanism for promoting audience deep understanding and risk awareness formation, and should be treated as a core dimension of platform content governance. The research results provide a quantitative basis for short video platforms to optimize the compliance of health science popularization content, emphasizing the core transmission role of knowledge absorption willingness in the effectiveness of health communication, and having important practical value for improving public health literacy (Table 13).

**Table 13:** Mediation analysis results - perceived content compliance.

Path	Estimate	95% CI [LL, UL]
Direct effect (c′)	0.145	[−0.022, 0.314]
Path a: Perceived content compliance → Knowledge absorption willingness	0.744***	[0.566, 0.957]
Path b: Knowledge absorption willingness → Health protective behaviors	0.759***	[0.606, 0.968]
Indirect effect (a × b): Perceived content compliance → Knowledge absorption willingness → Health protective behaviors	0.565**	[0.425, 0.754]
Total effect	0.71	—

c’ = direct effect; a x b = indirect effect; CI = confidence interval; LL = lower limit; UL = upper limit; * *p* < 0.05, ** *p* < 0.01, *** *p* < 0.001.

The indirect effect is based on 5,000 bootstrap samples; ** *p* < 0.01 (95% CI excludes zero).

#### 4.4.3 Analysis of the impact of perceived information disclosure compliance on health protective behaviors through knowledge absorption willingness

The mediation analysis results showed that the impact of perceived information disclosure compliance on health protective behaviors was primarily mediated through knowledge absorption willingness. The direct effect (c′ = 0.102, 95% CI [−0.046, 0.251]) did not reach statistical significance (confidence interval including zero), while the indirect effect was significant: path a (perceived information disclosure compliance →knowledge absorption willingness, *β* = 0.574 ***) and path b (knowledge absorption willingness →health protective behaviors, *β* = 0.747 ***) were both highly significant. The indirect effect value was 0.429 (95% CI [0.316, 0.576]), and the confidence interval did not include zero (*p* < 0.01), indicating that knowledge absorption willingness plays a fully mediating role between the two. The total effect was 0.531, indicating that the public’s perception of information disclosure compliance in health science popularizationdoes not directly enhance their health protective behaviors, but indirectly promotes the formation of risk awareness by increasing their willingness to actively acquire and internalize health knowledge. This finding highlights the bridge function of transparent and standardized information disclosure mechanisms in public health communication. Although it does not directly shape safety perception, it can effectively activate audience cognitive participation, thereby strengthening the effectiveness of science popularization. Therefore, short video platforms need to prioritize optimizing information disclosure compliance to enhance users’ willingness to absorb knowledge, thereby more effectively improving the level of public health protection. This has important practical guidance value for the formulation of health communication strategies (Table 14).

**Table 14:** Mediation analysis results - perceived information disclosure compliance.

Path	Estimate	95% CI [LL, UL]
Direct effect (c′)	0.102	[−0.046, 0.251]
Path a: Perceived information disclosure compliance → Knowledge absorption willingness	0.574***	[0.481, 0.675]
Path b: Knowledge absorption willingness → Health protective behaviors	0.747***	[0.531, 1.014]
Indirect effect (a × b): Perceived information disclosure compliance → Knowledge absorption willingness → Health protective behaviors	0.429**	[0.316, 0.576]
Total effect	0.531	—

c’ = direct effect; a x b = indirect effect; CI = confidence interval; LL = lower limit; UL = upper limit; * *p* < 0.05, ** *p* < 0.01, *** *p* < 0.001.

The indirect effect is based on 5,000 bootstrap samples; ** *p* < 0.01 (95% CI excludes zero).

#### 4.4.4 Analysis of the impact of perceived privacy protection compliance on health protective behaviors through knowledge absorption willingness

The mediation analysis results indicated that perceived privacy protection compliance had a significant positive impact on health protective behaviors, and this impact was achieved through both direct and indirect paths. The direct effect (c′ = 0.184, 95% CI [0.027, 0.361]) reached statistical significance (*p* < 0.05), indicating that when the public believes that the platform fully protects their privacy in health science popularization, it directly improves their level of health protection. The indirect effect was also significant: path a (perceived privacy protection compliance →knowledge absorption willingness, *β* = 0.617 ***) and path b (knowledge absorption willingness →health protective behaviors, *β* = 0.697 ***) were both highly significant. The indirect effect value was 0.430 (95% CI [0.319, 0.583], *p* < 0.01), indicating that knowledge absorption willingness plays a partial mediating role. The total effect was 0.614, highlighting that privacy protection compliance not only enhances user trust, but also further strengthens risk perception by stimulating their willingness to acquire knowledge. The research results emphasize that in health communication via short videos, a sound privacy protection mechanism is not only an ethical requirement, but also a key strategy to enhance health protective behaviors and information acceptance. Short video platforms need to consider both the direct effect of privacy protection compliance and the mediating role of knowledge absorption willingness, and improve the level of public health protection through a dual approach, which has important practical value for optimizing health communication strategies (Table 15).

**Table 15:** Mediation analysis results - perceived privacy protection compliance.

Path	Estimate	95% CI [LL, UL]
Direct effect (c′)	0.184*	[0.027, 0.361]
Path a: Perceived privacy protection compliance → Knowledge absorption willingness	0.617***	[0.501, 0.76]
Path b: Knowledge absorption willingness → Health protective behaviors	0.697***	[0.473, 0.938]
Indirect effect (a × b): Perceived privacy protection compliance → Knowledge absorption willingness → Health protective behaviors	0.43**	[0.319, 0.583]
Total effect	0.614	—

c’ = direct effect; a x b = indirect effect; CI = confidence interval; LL = lower limit; UL = upper limit; * *p* < 0.05, ** *p* < 0.01, *** *p* < 0.001.

The indirect effect is based on 5,000 bootstrap samples; ** *p* < 0.01 (95% CI excludes zero).

#### 4.4.5 Analysis of the impact of perceived communication behavior compliance on health protective behaviors through knowledge absorption willingness

The mediation analysis results showed that the impact of perceived communication behavior compliance on health protective behaviors was primarily mediated through knowledge absorption willingness. The direct effect (c′ = 0.038, 95% CI [−0.083, 0.162]) did not reach statistical significance (with a confidence interval of zero), while the indirect effect was significant: path a (perceived communication behavior compliance →knowledge absorption willingness, *β* = 0.550***) and path b (knowledge absorption willingness →health protective behaviors, *β* = 0.828 ***) were both highly significant. The indirect effect value was 0.455 (95% CI [0.345, 0.609]), and the confidence interval did not include zero (*p* < 0.01), indicating that knowledge absorption willingness plays a fully mediating role between the two. The total effect was 0.493, indicating that the public’s perception of compliance of health science communication behaviors on short video platforms (such as publication frequency, formal standardization, avoiding exaggeration, etc.) does not directly improve their level of health protection, but indirectly promotes the formation of risk awareness by enhancing their willingness to actively learn and internalize health information. This finding emphasizes that standardized communication behavior, although seemingly a behind-the-scenes operation, can effectively stimulate audience cognitive participation and is an important mechanism for improving the effectiveness of health communication. Therefore, platforms should strengthen the compliance management of the communication process of science popularization content, emphasizing the need to prioritize stimulating users’ willingness to absorb knowledge through compliant communication behaviors, in order to indirectly improve the level of public health protection and optimize public health cognitive outcomes. (Table 16).

**Table 16:** Mediation analysis results - perceived communication behavior compliance.

Path	Estimate	95% CI [LL, UL]
Direct effect (c′)	0.038	[−0.083, 0.162]
Path a: Perceived communication behavior compliance → Knowledge absorption willingness	0.55***	[0.426, 0.69]
Path b: Knowledge absorption willingness → Health protective behaviors	0.828***	[0.657, 1.035]
Indirect effect (a × b): Perceived communication behavior compliance → Knowledge absorption willingness → Health protective behaviors	0.455**	[0.345, 0.609]
Total effect	0.493	—

c’ = direct effect; a x b = indirect effect; CI = confidence interval; LL = lower limit; UL = upper limit; * *p* < 0.05, ** *p* < 0.01, *** *p* < 0.001.

The indirect effect is based on 5,000 bootstrap samples; ** *p* < 0.01 (95% CI excludes zero).

The results indicate that knowledge absorption willingness plays a key mediating role in all pathways and is the core psychological mechanism that connects platform compliance practices with public risk cognition.

Specifically, except for perceived privacy protection compliance and overall public perceived compliance, which showed significant direct and indirect effects (i.e., partial mediation), the other three dimensions all influenced health protective behaviors through a fully mediated pathway. That is, only when perception of compliance effectively stimulates users’ willingness to actively obtain, understand, and internalize health information can their level of health protection be improved. Among them, communication behavior compliance has the highest dependence on mediation (92.3%), highlighting the key role of timely forwarding of official information + rapid rumor debunking in stimulating knowledge absorption in public health emergencies. What is particularly noteworthy is that privacy protection compliance had a significant direct effect and can independently enhance public cognition through the compliance trust → safety perception pathway. In contrast, the direct effects of the other dimensions were not significant, and they required knowledge absorption willingness as a bridge to achieve indirect impact. This difference suggests that privacy protection compliance has a dual attribute of direct trust construction and indirect cognitive enhancement. In summary, platforms’ compliance construction in terms of content authority, information transparency, data security, and communication standardization is not only to meet regulatory requirements, but also a key prerequisite for activating public cognitive participation, and improving health literacy. In practice, a dual-track optimization strategy is proposed: for dimensions with significant direct effects such as privacy protection, efforts should focus on strengthening compliance labeling and transparency construction; for dimensions dominated by indirect effects, such as content and communication, interactive mechanisms (e.g., authoritative science pop-ups and rumor debunking columns) should be designed to enhance knowledge absorption willingness, ultimately achieving an efficient transformation from compliance to health protective behaviors.

## 5 Results and discussion

### 5.1 Research summary

This study examines the communication scenario of public health science and emergency prevention knowledge on digital health information platforms. Based on protection motivation theory and information adoption theory, it constructs a model linking public perceived compliance, knowledge absorption willingness, and health protective behaviors. The results show that perceived content compliance, perceived information disclosure compliance, perceived privacy protection compliance, and perceived communication behavior compliance are all positively associated with knowledge absorption willingness. Knowledge absorption willingness is positively associated with health protective behaviors and fully mediates the relationship between public perceived compliance and health protective behaviors. These findings suggest that platform compliance governance may improve the behavioral effectiveness of public health knowledge dissemination by strengthening users’ willingness to absorb and apply health information. The extended literature also shows that social media public health communication and TikTok-based community mitigation are important empirical settings for evaluating how short-video platforms translate public health guidance into everyday protective practice (32, 33). Systematic reviews of internet-based public health communication further indicate that communication effectiveness depends on channel design, message quality, and audience fit (34). Health misinformation remains a central boundary condition: recent systematic, bibliometric, and scoping reviews document its prevalence, persistence, and public health impact before and after COVID-19 (35–38).

The theoretical contributions of this study are reflected in three aspects. Firstly, it fills the research gap in the communication mechanism of health protection science popularization in specific scenarios of short videos. For the sub field of public health science popularization + emergency prevention and control knowledge, the independent and collaborative impacts of the four dimensions of perceived compliance have been systematically verified for the first time, filling the limitation of existing research that mainly focuses on general social media. Secondly, it clarifies the mediating value of knowledge absorption willingness by integrating the four dimensions of active attention–deep understanding–scenario application–sharing and communication into a mediating chain, thereby refining the theoretical logic underlying the formation of health protective behaviors. Thirdly, based on protection motivation theory and information adoption theory, it develops a four-dimensional perceived compliance scale adapted to short video platforms, solving the problems of inconsistent research dimensions and insufficient contextual suitability in existing research (2).

### 5.2 Practical implications

Based on the validated results of the public perceived compliance–knowledge absorption willingness–health protective behaviors model, combined with the positive impact of perceived content compliance, information disclosure compliance, privacy protection compliance, and communication behavior compliance on knowledge absorption willingness, as well as the fully mediating role of knowledge absorption willingness, this study proposes specific measures from three perspectives including platform governance, content creation, and regulatory coordination to promote the formation of public health protective behaviors: Evidence on short-video health knowledge adoption and online health misinformation detection suggests that platform governance should treat knowledge adoption, sharing, and misinformation identification as connected processes rather than separate tasks (39, 40).

#### 5.2.1 Platform governance: building a solid foundation for compliant communication and activating knowledge absorption willingness

In view of the core demands of perceived privacy protection compliance and communication behavior compliance, platforms need dual-track promotion of technical support and mechanism optimization. Technically, platforms can introduce health data encryption and de-identification measures and explain the privacy protection process in scenarios such as health questionnaire filling and health protection case sharing, so as to enhance the public’s privacy-security controllability perception (6). Optimize the algorithm recommendation logic, reduce the traffic weight of clickbait headline content and false health protection science popularization, and improve the recommendation priority for the content of authoritative sources such as the National Health Commission and the CDC, so that the compliant health protection content can accurately reach users and reduce the cost of information screening. Mechanistically, establish a four-dimensional review standard that incorporates scientific nature of content, information transparency, privacy protection measures, and communication standardization into the review system (1). Set up a fast track for official information release on public health emergencies, ensuring consistency between content and official statements, and avoiding information conflicts that weaken trust (24). Establish a 24-h science popularization content correction channel to promptly verify and provide feedback on violations reported by the public, forming a release–review–feedback–optimization loop. TikTok-based knowledge mobilization research shows that short-video platforms can support community-engaged public health knowledge circulation when content production is connected to audience needs and trusted intermediaries (41).

#### 5.2.2 Content creation: improving the quality of compliant communication and promoting deep transformation of knowledge

Focusing on the driving effect of perceived content and information disclosure compliance on knowledge absorption, creators need to work from two aspects: source authority and design adaptation. In terms of information sources, priority should be given to authoritative information sources such as CDC and tertiary hospitals. And the qualifications of producers, data sources and applicable populations should be clearly marked (5) to objectively express health risks. Avoid absolute and intimidating expressions, and use the positive emotional language (9) recommended by public health experts in Rao’s study, such as washing hands correctly can reduce the risk of infection by 80%. In terms of design, hierarchical creation is made for different groups of prior knowledge differences: for middle-aged and people over 65, professional terms are simplified with case + step; for the youth group, the data interpretation + frontier research module is added, which is in line with the research of prior knowledge and learning effect (14). At the same time, the scenario-based design is strengthened to simulate the life scenarios such as home epidemic prevention and sudden fever (15). Studies on willingness to share health misinformation videos, unverified health information sharing, and accuracy-nudge interventions indicate that creators should design content that makes verification easier and reduces impulsive sharing of unverified health claims (42–44).

#### 5.2.3 Regulatory coordination: improving the compliance governance system and strengthening multi-stakeholder collaboration

Focusing on the synergistic improvement of four-dimensional perceived compliance, a supervision–platform–authority linkage mechanism is constructed. The regulatory authorities need to issue special regulations to clarify that the content of health protection needs to be reviewed by the local CDC, the information needs to be marked with an update time, and the collection of irrelevant health data is prohibited. Regularly joint medical institutions to sample the health protection content of the platform, and implement graded punishment for illegal accounts. Platforms cooperate with the CDC to establish a health science popularization content library to provide certified health protection material templates. Authorities regularly carry out creator training, explain the key points of compliant communication (2), and improve the quality of content from the source. Evidence linking online misinformation to early vaccine hesitancy and research on information source, eHealth literacy, and misinformation interventions further support coordinated fact-checking, correction, and risk-communication mechanisms across regulators, platforms, and public health institutions (45–47).

### 5.3 Research limitations and future directions

Although this study clarifies the association between perceived compliance and health protective behaviors, several limitations remain. First, the cross-sectional survey design does not support strong causal inference. Second, the online non-probability sample may overrepresent users who are more familiar with digital platforms, and the low-frequency exposure pattern observed in the sample may affect the generalizability of the findings. Third, all variables were measured through self-reported questionnaires, which may introduce common method bias and social desirability bias. Fourth, the perceived privacy protection compliance dimension has lower internal consistency than the other dimensions, so the interpretation of this dimension should remain cautious. Future research should use longitudinal data, platform behavioral data, or experimental designs to test whether improvements in compliance governance produce measurable changes in health protective behaviors. Social media may also hinder learning about science when platform use fragments attention or exposes users to misleading COVID-19 information, so future studies should examine how short-video recommendation, user literacy, and content verification jointly shape learning outcomes rather than treating exposure as uniformly beneficial (48).

Future research can proceed in three directions. First, longitudinal tracking can be used to observe whether perceived compliance and health protective behaviors change over time. Second, platform-level indicators, such as content review records, source labeling, and privacy protection notices, can be combined with survey data to improve measurement validity. Third, group differences by age, education, place of residence, and exposure frequency should be examined to identify whether digital public health communication has unequal effects across population groups.

## 6 Conclusion

Digital health information platforms have become important carriers of public health science communication and emergency prevention knowledge. Their compliance governance is directly related to information accuracy, public trust, and the behavioral conversion of health knowledge. This study shows that perceived platform compliance can promote health protective behaviors through knowledge absorption willingness. For digital public health governance, this means that content accuracy, transparent disclosure, privacy protection, and standardized communication behavior should be treated as integrated policy concerns rather than isolated platform management issues.

## Data Availability

The raw data supporting the conclusions of this article will be made available by the authors, without undue reservation.
